# Integrative network analysis reveals organizational principles of the endocannabinoid system

**DOI:** 10.1186/s42238-026-00447-3

**Published:** 2026-05-13

**Authors:** Aanya Shridhar, Sugyan Mani Dixit, Anthony Torres, Reggie Gaudino

**Affiliations:** Office of the Vice Chancellor of Research, Cannabis Research Institute, 2201 W Campbell Park Drive, Room 115, Chicago, IL 60612 USA

**Keywords:** Endocannabinoid system, Biological network, Cannabinoid receptors, Cannabinoids, CB_1_, CB_2_, GPR55

## Abstract

**Background:**

The endocannabinoid system (ECS) is a complex signaling network that regulates diverse physiological processes, including pain, mood, metabolism, and immune response, through coordinated interactions among receptors, enzymes, and lipid-derived ligands. Although individual ECS components have been extensively studied, the integrated systems-level organization and structural dependencies of the ECS remain insufficiently characterized in a unified network context. Here, we present a computational, network-based systems analysis of the ECS that integrates protein–protein and protein–chemical interactions into a unified interaction framework, enabling the identification of components that occupy structurally prominent positions in the network, with potential relevance to the role of ECS in diverse physiological processes and therapeutic contexts.

**Methods:**

We constructed integrated ECS networks by combining experimentally validated protein–protein and protein–chemical interactions from multiple public databases. Network analyses were performed using centrality metrics, community detection algorithms, and targeted perturbations of highly ranked nodes to assess structural organization, modular architecture, and redistribution of topological influence.

**Results:**

Centrality analyses systematically identified nodes with high topological prominence across the ECS network. Canonical receptors cannabinoid receptor 1 (CB_1_) and cannabinoid receptor 2 (CB_2_) ranked consistently among the most influential nodes, while non-canonical components such as transient receptor potential vanilloid 1 (TRPV1), G-protein coupled receptor 55 (GPR55), peroxisome proliferator-activated receptor alpha (PPARα), cyclooxygenase-2 (COX-2), fatty acid amide hydrolase (FAAH), and diacylglycerol lipase alpha (DAGLα) also emerged as highly ranked nodes across multiple centrality measures. Closeness and eigenvector centrality further highlighted phytocannabinoids including cannabidiol (CBD), tetrahydrocannabivarin (THCV), and cannabidivarin (CBDV) as structurally well-connected components within the network. Community detection revealed a modular organization separating receptor-mediated signaling components from endocannabinoid metabolic processes, with clusters centered on CB_1_/CB_2_ signaling machinery and enzymes such as FAAH and diacylglycerol lipase beta (DAGLβ), which are associated with 2-arachidonoylglycerol (2-AG) turnover. Perturbation analyses demonstrated that removal of dominant hubs, particularly CB_1_, redistributed centrality and altered shortest-path structure, increasing the relative prominence of nodes such as CB_2_ and GPR55 while decreasing that of others such as DAGLβ and linoleoyl ethanolamide (LEA). These findings identify structurally influential and configuration-dependent nodes whose prominence becomes apparent through network-level analysis.

**Conclusion:**

By mapping the ECS as an integrated interaction network, this study provides a structural framework for understanding how receptors, enzymes, and ligands collectively shape ECS organization. Our results demonstrate that network analysis can identify structurally influential components within the ECS, highlighting nodes whose importance emerges from the overall network organization. The identification of highly ranked and perturbation-sensitive nodes offers a systematic basis for prioritizing underexplored components for hypothesis-driven experimental investigation and pharmacological study. More broadly, this work establishes a network-based foundation for expanding ECS modeling to incorporate additional molecular entities, interaction directionality, signaling dynamics, and tissue- or context-specific interactions, thereby informing future therapeutic strategies targeting the ECS and its interacting molecular pathways across diverse physiological processes and disease pathways.

**Supplementary Information:**

The online version contains supplementary material available at 10.1186/s42238-026-00447-3.

## Background

The endocannabinoid system (ECS) is a multifaceted regulatory network that maintains physiological homeostasis across diverse organ systems (Lu and Mackie 2021; Kunos et al. 2008; Sikyta et al. 2024). It is composed of endogenous ligands known as endocannabinoids, their cognate receptors, and the enzymes that mediate their synthesis and degradation (Lu and Mackie 2021; Schurman et al. 2019). Endocannabinoids are cannabinoids produced within the human body, in contrast to phytocannabinoids, which are derived from a number of plants, of which the best known is *Cannabis sativa* (Gülck and Møller 2020; Ribeiro et al. 2024; Chavez and D’Auria 2023), and synthetic cannabinoids, which are laboratory-engineered compounds designed to mimic or modulate the effects of natural cannabinoids for research and therapeutic applications (Lu and Mackie 2021; Schurman et al. 2019). Through the coordinated activity of these molecular components, the ECS influences a broad spectrum of biological processes, including pain perception, inflammation, memory, and mood regulation (Barrie and Manolios 2019; Campolongo and Trezza 2012; Simankowicz and Stępniewska 2025).

Two primary receptors define much of the functional landscape of the ECS. Cannabinoid receptor 1 (CB_1_, also called CNR1) is predominantly expressed in the brain and central nervous system, where it regulates mood, appetite, pain perception, and cognitive processes (Lu and Mackie 2021; Barrie and Manolios 2019; Campolongo and Trezza 2012; Simankowicz and Stępniewska 2025). Cannabinoid receptor 2 (CB_2_, also called CNR2) is primarily located in immune cells and peripheral tissues, where it regulates inflammatory and immune responses (Lu and Mackie 2021; Barrie and Manolios 2019; 2012; Campolongo and Trezza 2025). The ECS also interfaces with intracellular kinase signaling pathways that control cell survival, synaptic plasticity, and metabolic regulation. This extensive interconnectedness underscores how the ECS integrates with broader cellular signaling networks (Lu and Mackie 2021; Kunos et al. 2008; Sikyta et al. 2024; Schurman et al. 2019; Barrie and Manolios 2019; Campolongo and Trezza 2012; Simankowicz and Stępniewska 2025; Dörnyei et al. 2023; Haspula and Clark 2020). When the processes governing endocannabinoid synthesis and degradation become dysregulated, the resulting imbalance in “endocannabinoid tone” has been associated with a range of pathological conditions, including chronic pain, neurodegenerative disorders, metabolic syndromes, and cancer (Dörnyei et al. 2023; Haspula and Clark 2020; Russo 2016, 2001; Ross 2001).

The growing acknowledgment of the ECS’s role in human physiology has made it a key target for both basic and clinical research, particularly in understanding how endogenous and exogenous compounds can modulate its signaling (Howlett et al. 2002; Di Marzo et al. 2008; Pacher et al. 2006; Tudorancea et al. 2022). The active compounds delta-9-tetrahydrocannabinol (Δ^9^-THC) and cannabidiol (CBD), derived from medical cannabis, interact with and modulate the activity of cannabinoid receptors, specifically CB_1_ and CB_2_, influencing endocannabinoid signaling and therapeutic outcomes (Blebea et al. 2024; Mick and Douek 2024; Hoch et al. 2025). Specifically, CBD-rich formulations with minimal THC content are increasingly studied and prescribed for conditions such as epilepsy, anxiety, and inflammation (Blebea et al. 2024; Mick and Douek 2024; Hoch et al. 2025). Despite this rising interest, the ECS remains insufficiently characterized as a networked system, particularly in terms of how its molecular components interact and respond to changes (Lu and Mackie 2021). A systems-level understanding of these interactions is critical for identifying novel therapeutic targets and developing strategies that more precisely modulate ECS function (Bernabò et al. 2013).

In this study, we employed a computational systems biology framework to characterize the ECS as an integrated molecular interaction network. We constructed curated networks encompassing protein–protein and protein–chemical relationships within the system. Through network analysis, we identified structurally prominent nodes using multiple centrality measures and performed clustering analyses to delineate modular groupings of molecules with shared biological roles. To further examine network structure, we introduced targeted perturbations to assess topological robustness and to evaluate how the removal of highly connected nodes redistributes connectivity within the ECS interaction map. Collectively, this framework provides a systems-level structural representation of the ECS, offering insight into its organization, connectivity patterns, and potential applications in hypothesis-driven experimental and therapeutic exploration.

## Methods

### Data collection and network construction

To construct a comprehensive and well-supported representation of the ECS, we built upon a previously curated ECS interaction framework described by Bernabò et al. (2013), which assembled core ECS proteins and ligands through extensive curation of peer-reviewed experimental studies. In that study, molecular entities were selected based on reproducible experimental evidence from multiple independent reports, establishing a validated foundation of canonical ECS components. We used Bernabò et al. (2013) as a foundational reference because of its rigorous methodology and well-curated ECS components, allowing us to focus on the primary scope of this study, which is analyzing the network organization and topology of the ECS.

**Fig. 1:**
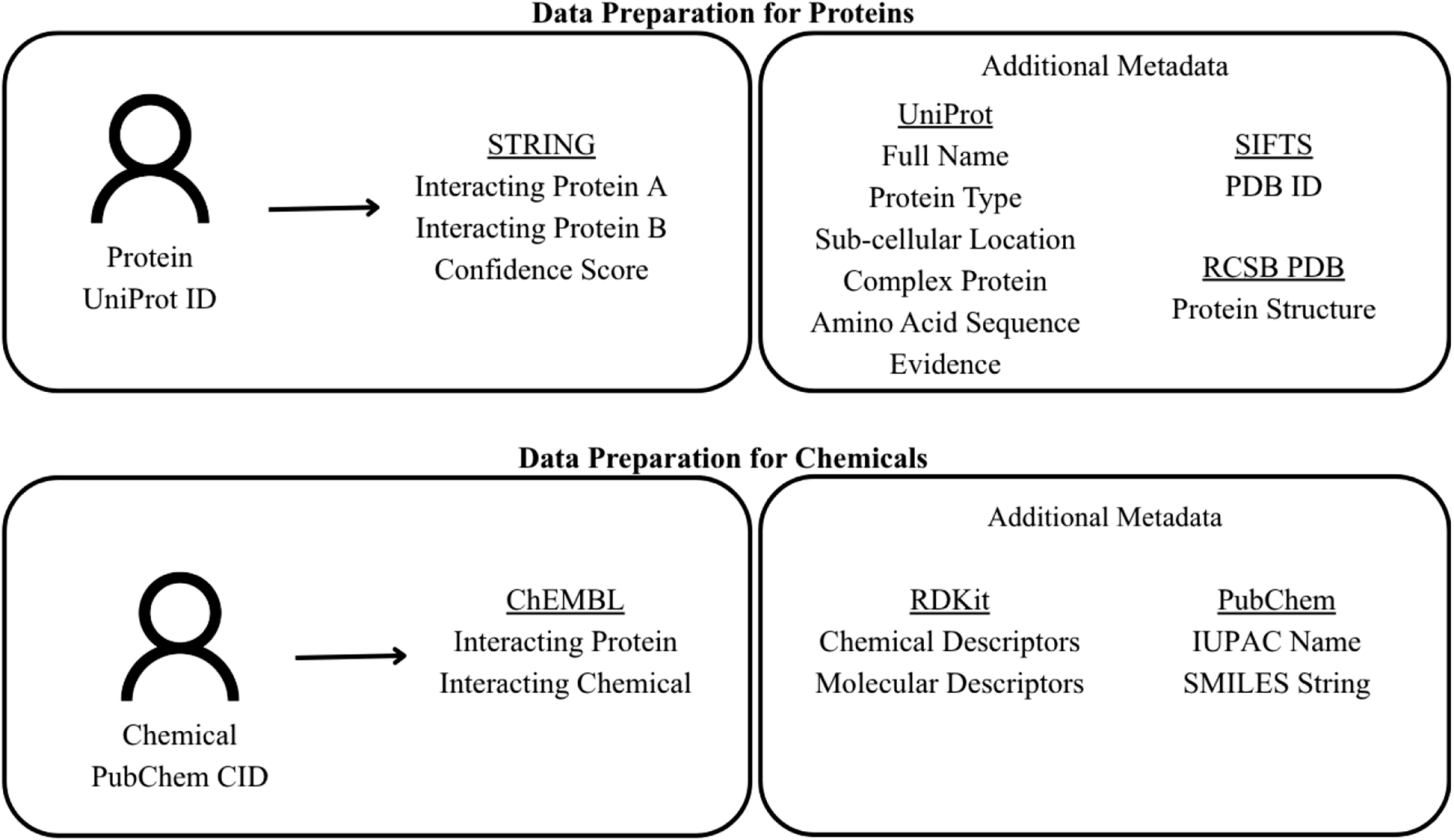
Data preparation pipeline for proteins and chemicals. Schematic depiction of the workflow used to compile protein–level and chemical–level data for network construction. Protein nodes (UniProt IDs) were annotated with interaction data from STRING, including interacting pairs and confidence scores. Additional metadata were integrated from UniProt, SIFTS, and RSCB PDB. Chemical nodes (PubChem CIDs) were annotated with interaction data from ChEMBL. Additional metadata were pulled from PubChem and calculated using RDKit

Using this curated set as a foundation, we compiled proteins, chemicals, protein–protein interactions (PPIs), and protein–chemical interactions (PCIs) from multiple public databases to generate an integrated interaction network (Fig. 1). In the resulting networks, nodes represent predefined ECS proteins or chemicals, and edges reflect experimentally supported interactions. All entities were annotated with structural and functional metadata to facilitate reproducibility and public access.

#### Proteins

A curated set of forty ECS-associated proteins was derived from the curated ECS framework described by Bernabò et al. (2013) and subsequently supplemented using UniProt and STRING database queries (Szklarczyk et al. 2023; Ahmad et al. 2025). This set includes canonical receptors CB_1_ and CB_2_, enzymes fatty acid amide hydrolase (FAAH), monoacylglycerol lipase (MAGL), and N-acyl-phosphatidylethanolamine phospholipase D (NAPE-PLD), and signaling partners. Core attributes, including amino acid sequence, sub-cellular localization, and complex membership, were retrieved from UniProt (2025). Structural mappings to the Protein Data Bank were obtained via SIFTS, and protein structures were extracted from RCSB PDB (Dana et al. 2019; Berman 2000). Protein–protein interactions were collected from STRING using a confidence threshold of 0.7 to reduce false positives (Szklarczyk et al. 2023). STRING confidence scores integrate multiple evidence types to estimate the likelihood of a true interaction, with values approaching one indicating higher confidence. All protein entries and protein–protein interactions were derived from human (*Homo sapiens*) data.

#### Chemicals

Forty ECS-relevant chemicals were similarly derived from prior literature (Bernabò et al. 2013) and included endocannabinoids anandamide (AEA) and 2-arachidonoylglycerol (2-AG), phytocannabinoids Δ^9^-THC and CBD, and selected eicosanoids. Chemical metadata, including IUPAC names and SMILES strings, were retrieved from the PubChem database (Kim et al. 2025). Molecular descriptors, including molecular weight, partial charges, and topological fingerprints, were calculated using RDKit to serve as metadata (Landrum et al. 2025). Protein-chemical interactions were defined as any experimentally supported association between a curated ECS chemical and a predefined ECS protein set. These interactions were retrieved from the ChEMBL database (Zdrazil et al. 2024). No explicit filtering was applied based on quantitative affinity or activity measures (e.g., Ki, Kd, EC50, or IC50), and interactions were not weighted. Due to the heterogeneity of assay types and activity annotations in ChEMBL, all supported interactions were treated as binary edges. Additionally, since interactions reported in ChEMBL may include multiple activity records derived from different assays or studies, each protein-chemical pair was represented as a single unique association in the network.

### Network construction and analysis

Three networks were constructed using NetworkX: a protein–protein only network, a protein–chemical only network, and a combined network containing all interactions (Hagberg et al. 2008). We modeled the networks as unweighted, undirected graphs where edges indicate the presence of experimentally supported interactions without encoding interaction strength or causal directionality. Network topology was quantified using centrality metrics, including betweenness, closeness, degree, and eigenvector. These measures identify nodes that are highly connected, influential, or serve as critical bridges within the ECS, providing insight into the system’s organizational hierarchy (Gómez 2019).

All centrality measures were computed using the NetworkX library, following its standard implementations (Hagberg et al. 2008). Degree centrality was calculated as the fraction of nodes in the network to which a given node is directly connected, normalized by the maximum possible degree. Betweenness centrality was computed as the proportion of shortest paths between all node pairs that pass through a given node. Closeness centrality was defined as the reciprocal of the average shortest path length from a node to all other reachable nodes, reflecting relative topological accessibility.

Eigenvector centrality was computed using the NetworkX implementation of the power iteration method, which estimates the dominant eigenvector of the graph's adjacency matrix. Centrality values were normalized to unit Euclidean norm, consistent with NetworkX's default behavior. In networks with disconnected components, eigenvector centrality is calculated using the entire graph. In these situations, the dominant eigenvector is mainly influenced by the largest connected component. As a result, nodes in smaller or isolated components often receive disproportionately lower centrality values because of their limited connectivity within the overall adjacency structure (Carreras et al. 2007; Van Mieghem 2016). Raw normalized eigenvector centrality values were used for all analyses, without logarithmic scaling or additional transformations.

### Network clustering

Functional modules within the ECS network were identified using the Louvain algorithm, which maximizes modularity by clustering nodes with preferential interactions (Li et al. 2024a). This approach highlights groups of proteins and chemicals that are likely to share biological functions or participate in coordinated processes.

### Network perturbations

Network robustness was evaluated by targeting the removal of specific nodes. Specifically, the nodes with the highest betweenness and degree centrality, namely the top one and top three nodes from each metric, were systematically removed to simulate the disruption of crucial, highly connected components. After each removal, the network was reassessed to determine the number of remaining interactions and connections and to evaluate changes in its overall structure. Centrality metrics were recalculated to identify shifts in the relative importance of the remaining nodes and to understand how the network reorganized in response to the loss of these critical elements (Bernabò et al. 2013; Pacher and Kunos 2013; Jaeger and Aloy 2012).

### Code and data availability

All data and code used in this study are available in a GitHub repository at https://github.com/aanyashridhar/ECS-Network.

## Results

**Fig. 2:**
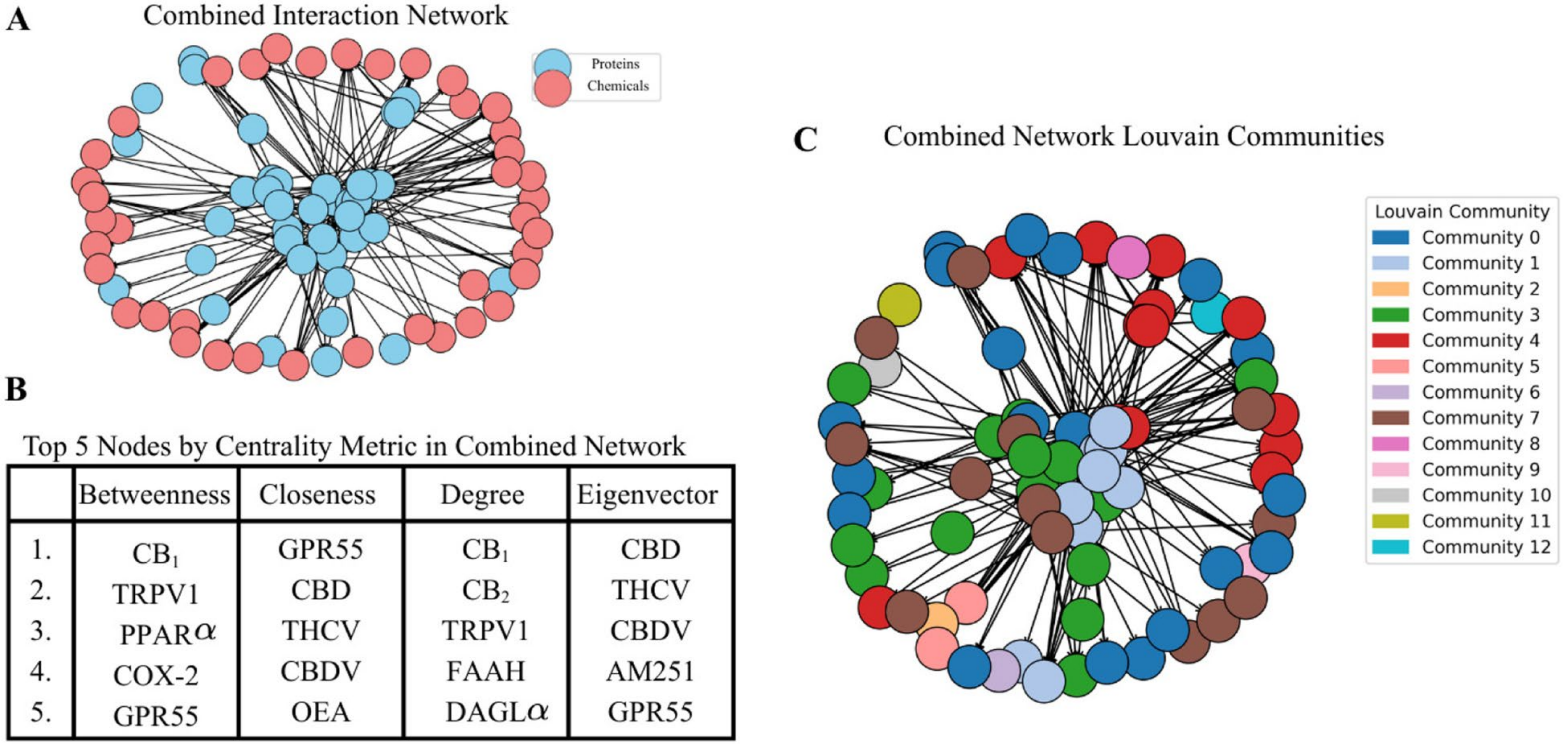
Combined interaction network and node characterization.** A** Visualization of the integrated network showing proteins (blue) and chemicals (red) connected through documented interactions. The network has 80 nodes and 218 edges. The network layout represents the network’s topological organization based on connectivity and does not reflect signaling direction, cellular location, or dynamic activity. **B** Top five nodes ranked by centrality measures in the combined network. Results highlight distinct sets of influential nodes across betweenness, closeness, degree, and eigenvector centrality metrics, underscoring multiple dimensions of node importance. Full rankings and centrality metric calculations can be found at https://github.com/aanyashridhar/ECS-Network. **C** Louvain community detection applied to the combined network, partitioning the nodes (denoted by distinct colors) into modular clusters based on interaction density, which highlights potential functional or mechanistic groupings within the ECS network

The combined ECS network was constructed from the curated ECS component from Bernabò et al. (2013) as a foundation and expanded with protein–protein and protein-chemical interactions from STRING and ChEMBL databases. This unified network comprised 80 nodes and 218 edges and served as the primary framework for subsequent analyses (Fig. 2a, Tables S1-S4). Edges were treated as unweighted and non-directional, such that the interactions represent the presence of an association without encoding interaction strength or causal directionality. In this network, proteins occupy the central core, while chemicals are positioned toward the periphery. This structural organization reflects the biological architecture of the ECS, where proteins act as molecular hubs mediating signaling and metabolic processes, and chemicals serve as ligands or substrates that connect through these central protein nodes. The resulting topology suggests that the ECS is organized around a dense protein interaction core that enables diverse chemical inputs to converge on shared molecular pathways.

For comparison, the protein–protein and protein–chemical subnetworks, analyzed separately, contained 40 nodes and 92 edges, and 80 nodes and 126 edges, respectively. These subnetworks are shown in Figures S1–S4 and Tables S1–S4, and provide additional insight into the distinct interaction patterns that contribute to the overall connectivity of the ECS.

### Network centrality analysis reveals the hierarchical organization of the ECS and provides insight into connectivity and influence patterns

To understand the structural organization of the ECS network, we quantified key centrality measures that reflect node influence and connectivity (Fig. 2b, Tables S12-S13). The analysis revealed distinct patterns in how proteins and chemicals occupy topological positions within the combined network.

To evaluate the stability of these centrality metrics with respect to network composition, we performed a sensitivity analysis by removing the ten lowest-degree nodes and recalculating the centrality measures for the remaining network. This alteration had a minimal effect on the relative rankings of the highly connected nodes (Table S5), suggesting that the centrality patterns are stable and not influenced by marginal or sparsely connected components.

The five nodes with the highest betweenness centrality (Gómez 2019) in the combined network were CB_1_, transient receptor potential vanilloid 1 (TRPV1), peroxisome proliferator-activated receptor alpha (PPARα), cyclooxygenase-2 (COX-2), and G-protein coupled receptor 55 (GPR55), in order of rank (Fig. 2b). High betweenness centrality indicates that these nodes occupy positions through which many shortest paths traverse, reflecting their topological role as connectors within the ECS network. As one of the two primary cannabinoid receptors, CB_1_ connects to numerous cannabinoids and signaling components, serving as a central bridge in the ECS (Howlett et al. 2002; Mackie 2008). The second-highest ranked node, TRPV1, though not a canonical cannabinoid receptor, is a ligand-gated ion channel that mediates pain perception, inflammation, and thermoregulation (Rosenbaum and Simon 2007; Palazzo et al. 2008; Cui et al. 2006). It is strongly activated by cannabinoids, positioning it as a key integrative node within the ECS. The third-ranked PPARα is a nuclear receptor linking cannabinoid signaling to metabolic regulation (Iannotti and Vitale 2021). The fourth-ranked enzyme, COX-2, converts endocannabinoids into prostaglandins, occupies a position connecting inflammatory and pain-related pathways (Fowler 2007). Finally, GPR55, ranked fifth, is a G-protein coupled receptor (GPCR) that functions as a major non-cannabinoid receptor, reflecting its topological prominence in ECS connectivity (Ryberg et al. 2007).

The five nodes with the highest closeness centrality (Gómez 2019) in the combined network were GPR55, CBD, tetrahydrocannabivarin (THCV), cannabidivarin (CBDV), and oleoylethanolamide (OEA), in descending order (Fig. 2b). High closeness centrality indicates that these nodes occupy positions from which they can reach many other nodes in the network through relatively few steps, reflecting their topological centrality within the ECS network. GPR55 connects to multiple cannabinoids and receptors, resulting in short path lengths across the network (Ryberg et al. 2007). CBD, THCV, and CBDV follow closely, consistent with their interactions across diverse ECS targets, including CB_1_, CB_2_, TRPV channels, and GPCRs (Blebea et al. 2024). The fifth-ranked OEA, an endocannabinoid-like lipid, shares biosynthetic and signaling pathways with canonical endocannabinoids but does not bind directly to the cannabinoid receptors CB_1_ or CB_2_ (Barrie et al. 2022).

Nodes ranked highest by degree centrality (Gómez 2019) in the combined network were CB_1_, CB_2_, TRPV1, FAAH, and diacylglycerol lipase alpha (DAGLα), in order (Fig. 2b). High degree centrality indicates that these nodes are highly connected hubs within the network, reflecting their broad engagement across ECS components and their structural importance for maintaining network connectivity. CB_1_ and CB_2_, the two canonical cannabinoid receptors, exhibit extensive connectivity across both protein and chemical domains (Howlett et al. 2002; Mackie 2008). TRPV1, again, emerges as a highly connected integrator of cannabinoid and non-cannabinoid signaling (Rosenbaum and Simon 2007; Palazzo 2008; Cui et al. 2006). The fourth-ranked FAAH, a degradation enzyme (Deutsch et al. 2002), and the fifth-ranked DAGLα, a biosynthetic enzyme (Reisenberg et al. 2012), occupy central positions linking metabolic processes with other network components, highlighting their topological prominence and potential importance within the ECS network.

Finally, nodes ranked highest by eigenvector centrality (Gómez 2019; Carreras et al. 2007; Van Mieghem 2016) in the combined network were CBD, THCV, CBDV, AM251, and GPR55, in descending order (Fig. 2b). High eigenvector centrality indicates that these nodes are not only well-connected themselves but also closely linked to other influential nodes, emphasizing their role in shaping highly interactive subnetworks within the ECS. The prominence of CBD, THCV, and CBDV highlights the influence of phytocannabinoids within the network, as they engage several central receptors and enzymes (Blebea et al. 2024). AM251, a synthetic CB_1_ antagonist, ranks fourth due to its strong association with the CB_1_ receptor, reflecting its frequent use in receptor-targeting studies (Rodgers et al. 2005). GPR55 was also ranked among the top five, consistent with its high connectivity within the ECS interaction network (Ryberg et al. 2007).

### Network clustering analysis uncovers the modular architecture of the ECS and identifies biologically meaningful groupings of receptors, enzymes, and ligands

Clustering analysis identified thirteen distinct communities within the ECS network (Fig. 2c, Tables S6-S7). The largest community consisted of six proteins and fourteen chemicals, while the smallest communities included only a single node (Tables S6-S7). Although these isolated nodes were formally classified as communities by the algorithm, they do not represent meaningful biological groupings. The overall modularity of 42.77% (Table S7) falls within the expected range for biological networks (typically 30–70%) (Sah et al. 2014), indicating a balance between connectivity and modular organization.

Among the identified clusters, two communities were particularly informative. The first and largest, designated community 0 (Table S6), captured the core signaling architecture of the ECS. This cluster included the two primary cannabinoid receptors (CB_1_ and CB_2_ (Haspula and Clark 2020; Howlett et al. 2002; Mackie 2008)), PLC enzymes (phospholipase C epsilon 1 (PLCε1) and phospholipase C beta 1 (PLCβ1) (Bunney and Katan 2011)), signaling partners (inhibitory Guanine nucleotide-binding protein G alpha subunits i1–3 (Gαi1-3) (Villaseca et al. 2022)), numerous synthetic cannabinoids (WIN55,212–2 (Price et al. 2009), WIN55,212–3 (Price et al. 2009), JWH-018 (Huffman et al. 1994), JWH-073 (Huffman et al. 1994), JWH-133 (Huffman et al. 1994), CP55,940 (Ortega et al. 2015), SR141716A (Rinaldi-Carmona et al. 1994), SR144528 (Rinaldi-Carmona et al. 1994), and AM251 (Rodgers et al. 2005)), and endocannabinoid AEA (Devane et al. 1992; Lu and Mackie 2016). The Gαi proteins represent intracellular signaling mediators in the ECS, while synthetic cannabinoids and AEA illustrate the diversity of ligand–receptor interactions within the system. This cluster can be viewed as the cannabinoid receptor group, showing the cannabinoid receptors and their immediate effectors clustering strongly together. This organization reinforces the biological validity of the network and demonstrates that its modular structure captures known ECS signaling relationships.

The second notable cluster, community 1 (Table S6), which is the fifth largest community, centered on enzymes and molecules involved in the metabolism of endocannabinoids. This community contained degradation enzymes (FAAH (Deutsch et al. 2002), MAGL (Dinh et al. 2002), α/β-hydrolase domain-containing protein 6 (ABHD6) (Marrs et al. 2010; Savinainen et al. 2012), and α/β-hydrolase domain-containing protein 12 (ABHD12) (Marrs et al. 2010; Savinainen et al. 2012)), synthesis enzymes (NAPE-PLD (Tevosian et al. 2023), DAGLα (Reisenberg et al. 2012), and diacylglycerol lipase beta (DAGLβ) (Reisenberg et al. 2012)), non-canonical receptor GPR55 (Ryberg et al. 2007; Tudurí et al. 2017), transporter (fatty acid-binding protein 7 (FABP7) (Kaczocha et al. 2009)), and endocannabinoid 2-AG (Lu and Mackie 2016). Together, these nodes represent the metabolic group of the ECS, highlighting the tightly coupled nature of endocannabinoid synthesis, degradation, and transport. The inclusion of GPR55 within this community further underscores the functional integration of metabolic and signaling processes that maintain ECS homeostasis.

Beyond these two modules, additional multi-node communities (containing more than two nodes) also reflected distinct functional roles. Community 3 grouped nuclear receptors (PPARα, PPARδ, and PPARγ) (Iannotti and Vitale 2021), lipid-binding proteins (Fatty acid-binding protein 1 (FABP1), FABP3, and FABP5) (Elmes et al. 2019; Glaser et al. 2023), signaling regulators (AKT1 (serine/threonine protein kinase), β-arrestin-1, and G protein-coupled receptor kinase 2 (GRK2)) (Reiter and Lefkowitz 2006; Vroon et al. 2006; Blando et al. 2022), phytocannabinoids (Δ^9^-THC and Δ^8^-THC) (Ledvina et al. 2023; Ligresti et al. 2016), and lipid mediators (OEA and prostaglandin D2 (PGD2)) (Bestard-Escalas et al. 2026; Kolousek et al. 2023; Rahman et al. 2021; Wójcik et al. 2020). This organization suggests a transcriptional and signaling-integration module linking lipid-derived molecules and phytocannabinoids to nuclear receptor activation and downstream regulatory pathways (Nguyen et al. 2012; Huang et al. 2018; Schroeder et al. 2016; Iannotti and Vitale 2021; Pistis and Melis 2010). Community 4 consisted of TRP channels (TRPV1–4) (Storozhuk and Zholos 2018; Marzo and Petrocellis 2010; De Petrocellis et al. 2011; Rossi et al. 2011; De Petrocellis et al. 2012), several phytocannabinoids (cannabinol (CBN), cannabichromene (CBC), cannabigerol (CBG), THCV, and CBDV) (Zhang et al. 2023a; Jurga et al. 2024; Rezende et al. 2023), endogenous lipid mediators (N-arachidonoyl dopamine (NADA), N-arachidonoyl glycine (NAGly), and palmitoylethanolamide (PEA)) (Greco et al. 2022; Bradshaw et al. 2009; Deveci et al. 2020; Grabiec et al. 2012), and N-acylethanolamine-hydrolyzing acid amidase (NAAA) (Piomelli et al. 2020; Zhao et al. 2007), reflecting a non-canonical cannabinoid signaling axis centered on TRP-mediated sensory and inflammatory modulation (Barrie and Manolios 2017; Șerban et al. 2025). Lastly, community 7 comprised enzymes involved in arachidonic acid metabolism (COX-2, arachidonic acid lipoxygenase 5 (ALOX5), ALOX12, and ALOX15) (Kolousek et al. 2023; Ivanov et al. 2021; Wu et al. 2018; Tallima 2021; Wang et al. 2021; Zhang et al. 2023b), their substrate arachidonic acid (AA), and downstream lipid mediators (prostaglandin 2 (PGE2), prostaglandin A2 (PGA2), leukotriene B4 (LTB4), 12-hydroxyeicosatetraenoic acid (12-HETE), and 15-HETE) (Wang et al. 2021; Zhang et al. 2023b; Chen et al. 2023; Yamaguchi et al. 2022; Biernacki and Skrzydlewska 2025), representing a module associated with eicosanoid and inflammatory lipid metabolism (Biernacki and Skrzydlewska 2025; Rouzer and Marnett 2011). Together, these communities illustrate that the ECS network organizes into discrete yet interconnected topological modules spanning receptor signaling, lipid metabolism, transcriptional regulation, and inflammatory pathways.

### Network perturbation analysis illustrates how removal of key nodes reshapes ECS connectivity, revealing patterns of topological reorganization within the interaction network

A total of four perturbations were performed for each network, including the removal of the top one node by betweenness centrality, the top three nodes by betweenness centrality, the top one node by degree centrality, and the top three nodes by degree centrality. The protein–chemical only network was limited to two perturbations because betweenness centrality relies on shortest path calculations between nodes, which are of limited interpretability in this bipartite network, where chemicals only connect to proteins, allowing only degree-based removals. Across all networks, this approach produced a total of ten perturbations (Table 1). After each perturbation, the change in the number of edges before and after node removal was calculated as a high-level indicator of network impact. Perturbation by betweenness centrality in the protein–protein only network produced the most pronounced effect, with a 40.22% reduction in edge count. In general, removing the top one node, regardless of metric, decreased edge count by approximately 14–16%, whereas removing the top three nodes caused a larger reduction of roughly 34–40%. Notably, CB_1_ was removed at least once in all networks, regardless of whether it was identified as a top-ranked node based on betweenness or degree centrality, underscoring its structural importance within the ECS.

**Table 1:**
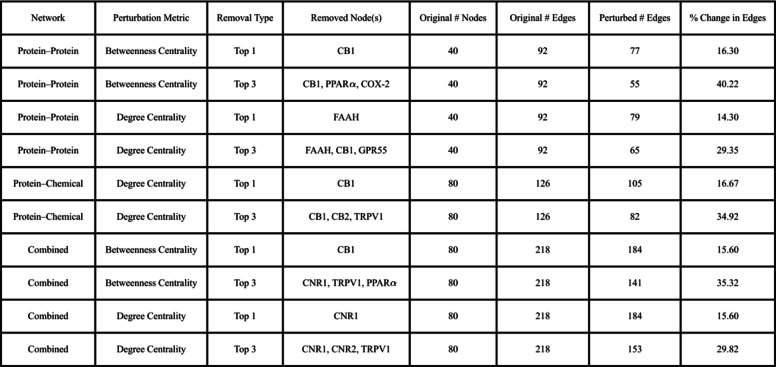
Network perturbation analysis across protein–protein, protein–chemical, and combined ECS networks. Summary of robustness simulations following targeted removal of highly central nodes (top one or top three rankings in betweenness or degree centrality). For each perturbation, the table shows the number of original nodes and edges, number of edges after perturbation, and percent change in edge count

**Fig. 3:**
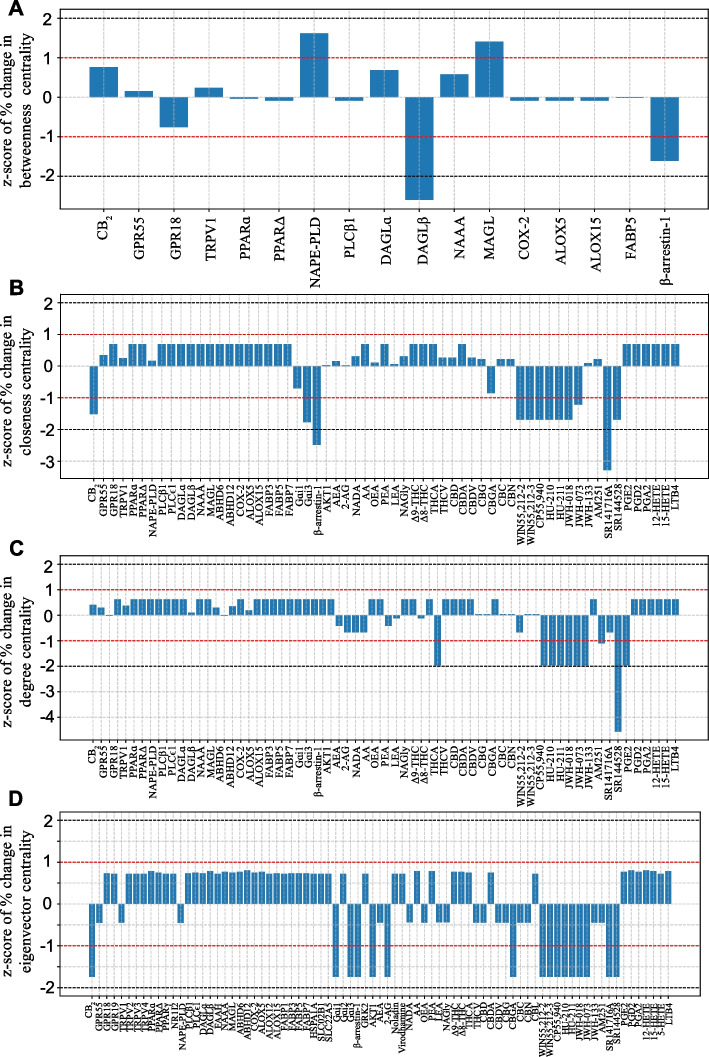
Network perturbation effects on node centrality following removal of the top-ranked node by betweenness centrality (top 1). Bar plots depict standardized changes (z-scores) in node centrality metrics after removal of the top-ranked node. Plots **A-D** illustrate changes in betweenness, closeness, degree, and eigenvector centrality, respectively. The red and black horizontal dotted lines denote absolute z-scores of 1 and 2, respectively

**Fig. 4:**
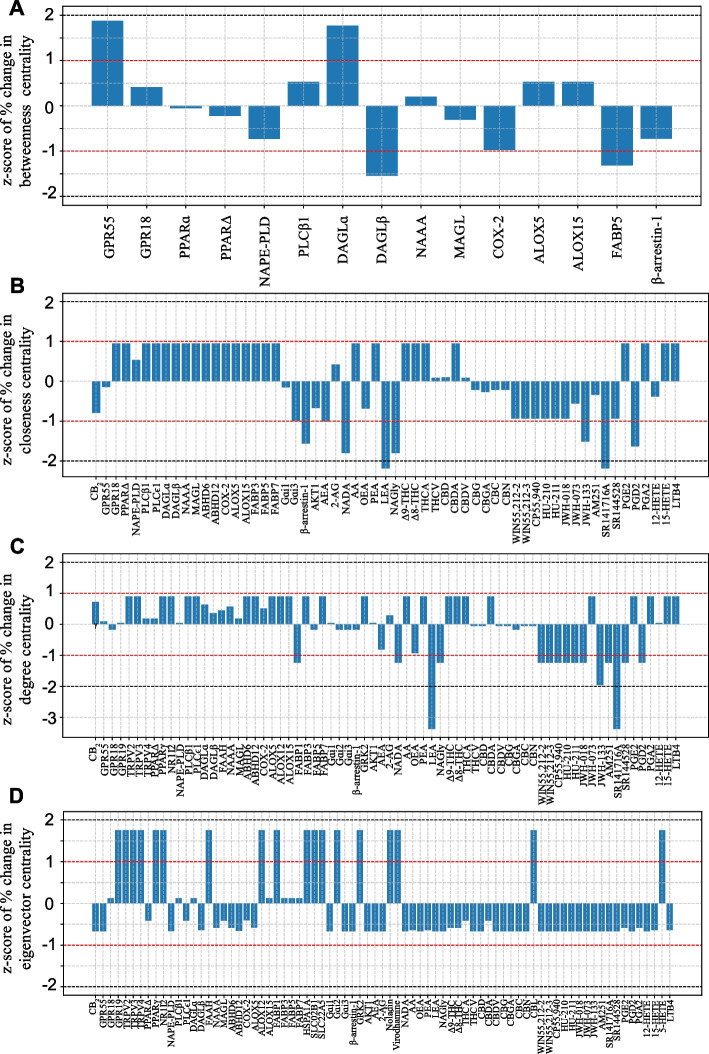
Network perturbation effects on node centrality following removal of the top 3 nodes by betweenness centrality (top 3). Bar plots depict standardized changes (z-scores) in node centrality metrics after removal of the top 3 nodes. Plots **A-D** illustrate changes in betweenness, closeness, degree, and eigenvector centrality, respectively. The red and black horizontal dotted lines denote absolute z-scores of 1 and 2, respectively

**Fig. 5:**
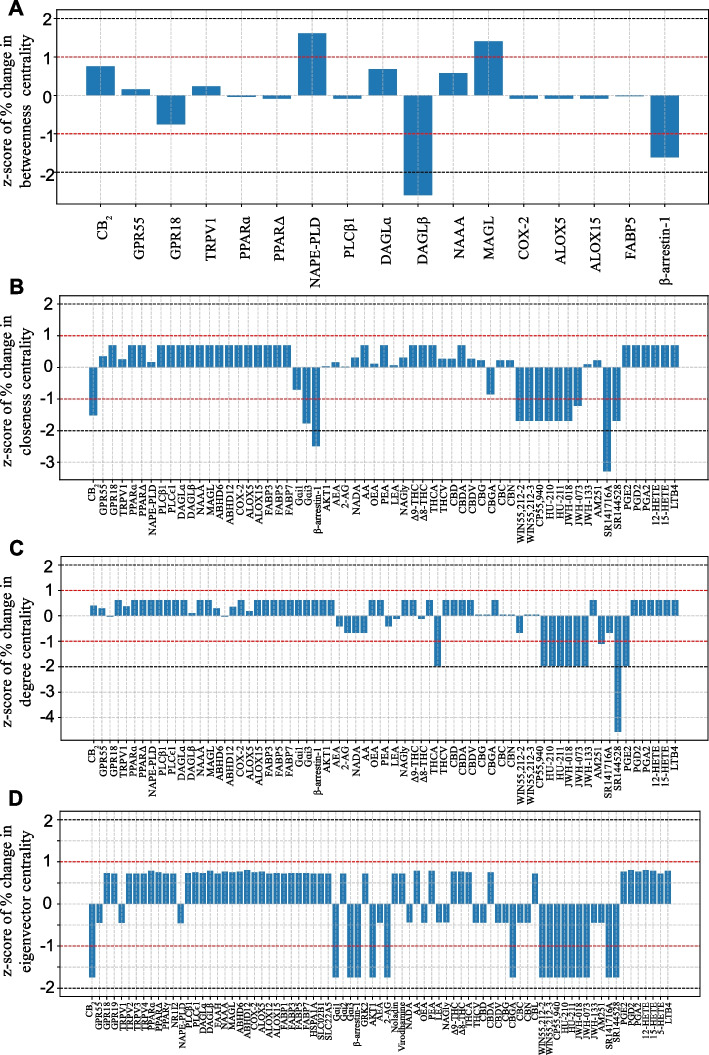
Network perturbation effects on node centrality following removal of the top-ranked node by degree centrality (top 1). Bar plots depict standardized changes (z-scores) in node centrality metrics after removal of the top-ranked node. Plots **A-D** illustrate changes in betweenness, closeness, degree, and eigenvector centrality, respectively. The red and black horizontal dotted lines denote absolute z-scores of 1 and 2, respectively

**Fig. 6:**
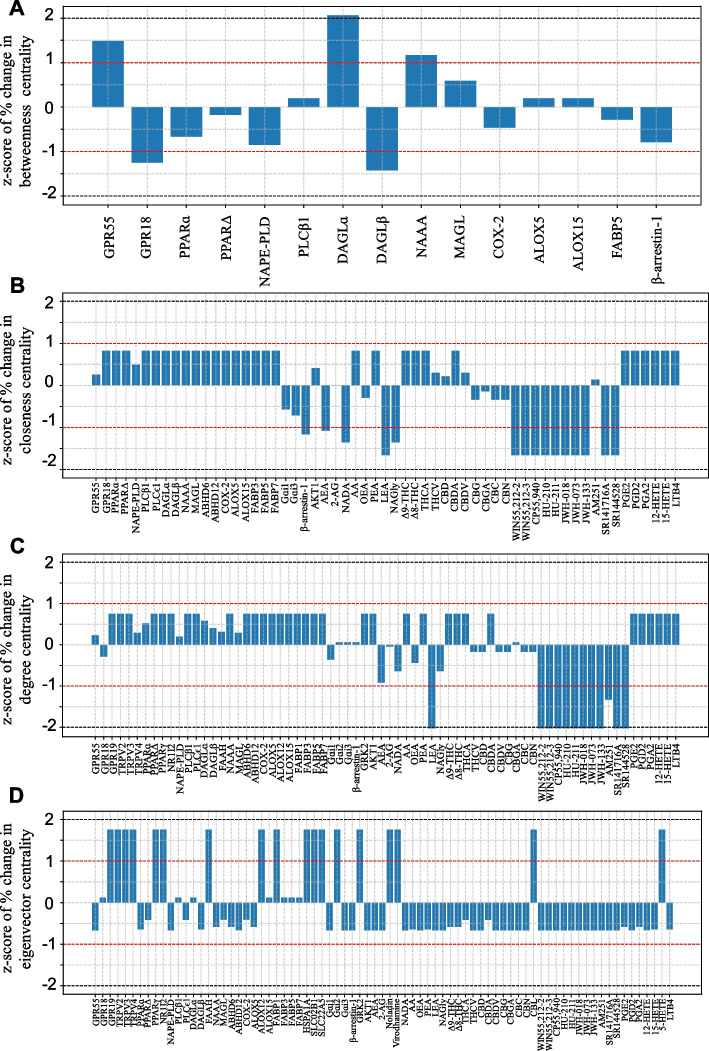
Network perturbation effects on node centrality following removal of the top 3 nodes by degree centrality (top 3). Bar plots depict standardized changes (z-scores) in node centrality metrics after removal of the top 3 nodes. Plots **A-D** illustrate changes in betweenness, closeness, degree, and eigenvector centrality, respectively. The red and black horizontal dotted lines denote absolute z-scores of 1 and 2, respectively

To evaluate how influential nodes shape network organization, we performed targeted perturbations informed by centrality rankings. Nodes were selected for removal based on their rankings in betweenness and degree centrality, which capture shortest-path mediation and connectivity, respectively. For each perturbation, key centrality measures, including betweenness, closeness, degree, and eigenvector centrality, were recalculated to assess shifts in network structure. The percent change in each metric was standardized relative to the distribution of changes across all nodes. Perturbations involving the removal of the top one or top three nodes by betweenness centrality (Figs. 3 and 4) and degree centrality (Figs. 5 and 6) were used to examine how hub disruption alters network topology.

Removal of highly connected or central nodes produced marked redistribution of centrality across the remaining network. For example, in the absence of CB_1_, receptors CB_2_ (Mackie 2008) and GPR55 (Ryberg et al. 2007) exhibited increases in centrality (Figs. 3a, 4a, 5a, and 6a), reflecting their increased structural prominence under the altered network configuration. Conversely, degradation enzymes such as DAGLβ (Reisenberg et al. 2012) showed decreases in betweenness centrality by over two standard deviations (Figs. 3a and 5a), indicating reduced positional influence following CB_1_ removal. Similarly, the endocannabinoid-like lipid LEA (Barrie et al. 2022) exhibited a drop in closeness centrality by nearly two standard deviations following the removal of the top-ranked nodes (Figs. 4b and 6b), reflecting decreased topological accessibility within the perturbed network. These shifts indicate that node centrality is sensitive to hub disruption and highlight components whose structural roles depend on the presence of dominant connectors.

Synthetic cannabinoids, including WIN55,212–2 (Price et al. 2009), WIN55,212–3 (Price et al. 2009), JWH-018 (Huffman et al. 1994), JWH-073 (Huffman et al. 1994), JWH-133 (Huffman et al. 1994), HU-210 (Ottani and Giuliani 2001), HU-211 (Ottani and Ortega Giuliani 2001), CP55,940 (Ortega et al. 2015), SR141716A (Rinaldi-Carmona et al. 1994), and SR144528 (Rinaldi-Carmona et al. 1994), exhibited significant decreases in network metrics following node removal (Figs. 3c, 4c, 5c, and 6c). For example, removing the top ranked node based on both betweenness and degree centrality caused the degree centrality values of these compounds to decrease significantly, as shown in Figs. 3c and 5c. Several synthetic cannabinoids dropped in degree centrality by nearly two standard deviations, with SR141716A decreasing by over four standard deviations (Figs. 3c and 5c). These results reflect the strong topological dependence of these ligands on highly connected receptor nodes within the current interaction map.

Finally, a comparison of eigenvector centrality changes following the removal of the top one versus the top three nodes by degree centrality revealed a redistribution of influence (Figs. 3d, 4d, 5d, and 6d). Removal of a single top node led to substantial decreases in centrality across many nodes, consistent with reduced cohesion around a dominant hub. In contrast, removing the top three nodes produced positive shifts in eigenvector centrality, indicating a reallocation of structural prominence within the network. This pattern suggests that, in the absence of dominant hubs, alternative nodes assume higher relative centrality within the perturbed configuration. These results should be interpreted as structural shifts within a static network rather than evidence of functional compensation or biological resilience.

## Discussion

The ECS network presented here was constructed from a manually curated component set grounded in the study by Bernabò et al. (2013) and subsequently expanded using established databases. The network constructed in this study incorporates protein–protein and protein-chemical interactions retrieved from STRING and ChEMBL databases, respectively, building on the curated ECS components from Bernabò et al. (2013). While this approach provides biological relevance and conceptual consistency, it necessarily reflects the current state of ECS research. As with any literature-informed curation strategy, the resulting network may be influenced by publication bias, uneven experimental characterization, and database coverage (Wodak et al. 2013; Snider et al. 2015; Schaefer et al. 2015; Gillis et al. 2014; Prakash et al. 2007; Schnoes et al. 2013). Proteins and chemicals that are more extensively characterized may exhibit higher connectivity within the network, reflecting both their biological importance and the depth of experimental investigation available in current literature and databases (Wodak et al. 2013; Snider et al. 2015; Schaefer et al. 2015; Gillis et al. 2014; Schnoes et al. 2013). Accordingly, this network should not be considered a complete or exhaustive representation of the ECS, but rather a structured approximation based on currently available data. Nevertheless, the integrated dataset provides a sufficiently robust foundation for examining the organizational and topological properties of the ECS at a systems level.

Protein–protein interactions were identified among the ECS proteins based on the components from Bernabò et al. (2013) and expanded with STRING interactions filtered by a specified confidence threshold to prioritize higher-confidence associations. STRING aggregates evidence from curated experimental data, database annotations, co-expression analyses, text mining, and computational inference (Szklarczyk et al. 2023). Although applying a confidence cutoff reduces the inclusion of lower-support interactions, the resulting PPI layer may still contain indirect or functionally inferred associations that do not necessarily correspond to direct physical binding events (Wodak et al. 2013; Snider et al. 2015; Gillis et al. 2014). Thus, the PPI component of the network represents high-confidence literature-supported and computationally integrated relationships within the curated ECS protein set, rather than an exclusively structure-resolved interactome (Wodak et al. 2013; Snider et al. 2015; Gillis et al. 2014; Schnoes et al. 2013). Emerging large-scale and structure-informed interaction datasets, including 3D interaction mapping and deep learning–based PPI prediction frameworks (Baranwal et al. 2022; Wu et al. 2023a; Patel et al. 2017; Xiong et al. 2025; Fang et al. 2021), may further refine ECS network resolution in future work by distinguishing direct molecular interfaces and expanding interaction coverage.

Protein–chemical interactions in the present network reflect curated literature-supported associations rather than quantitative pharmacological parameters (Bernabò et al. 2013). Because ChEMBL integrates diverse assay types, including binding and functional studies conducted across varying experimental contexts, individual interactions may differ substantially in affinity, efficacy, and physiological relevance (Zdrazil et al. 2024). For example, ChEMBL captures interactions derived from assays such as ligand binding affinity measurements (e.g., Ki or Kd), functional activity assays (e.g., EC50 or IC50) and cellular response studies conducted under various experimental conditions. By representing these heterogeneous measurements as binary edges, the current framework emphasizes structural connectivity over biochemical precision. While this abstraction enables global topological analysis, it does not distinguish between high-affinity canonical interactions and weaker or context-dependent associations. Incorporating affinity-weighted edges or multilayer representations would allow future studies to more directly model pharmacological strength and signaling specificity (Zheng et al. 2019; Berenstein et al. 2016; Zhang et al. 2016; Jiang et al. 2022; Cheng et al. 2012).

In addition, all interactions were modeled as unweighted and non-directional edges. This abstraction enables global topological analyses but does not capture interaction strength, binding affinity, temporal dynamics, or signaling directionality (Bosque et al. 2014; Martínez et al. 2023; Gursoy et al. 2008; Wuchty 2006). Consequently, measures such as degree or betweenness centrality reflect structural prominence within the curated interaction framework rather than quantitative biochemical influence or causal regulatory flow. This simplification of interactions in the network limits the extent to which signaling dynamics, interaction strength, and directional effects can be inferred, and highlights the importance of integrating additional data sources to develop more detailed and context-specific ECS models. Future extensions incorporating weighted edges, context-specific interaction strengths, or directed signaling relationships could provide a more mechanistic representation of ECS dynamics (Berenstein et al. 2016; Zhang et al. 2016; Jiang et al. 2022; Cheng et al. 2012; Wuchty 2006; Nasser et al. 2011; Rush and Repsilber 2018; Chen et al. 2021; Li et al. 2024b). Despite these limitations, the current network offers a systematic and reproducible framework for interrogating ECS organization at a systems level.

Our network-based analysis demonstrates how centrality measures can systematically identify nodes with high topological prominence within the ECS interaction network. Distinct patterns emerged across various metrics. Betweenness and degree centrality highlighted both canonical and non-canonical proteins, including CB_1_, TRPV1, PPARα, COX-2, FAAH, and DAGLα, indicating their roles as hubs or connectors within the network. In contrast, closeness and eigenvector centrality highlighted chemicals such as CBD, THCV, and CBDV, demonstrating their extensive network reach and connectivity (see Fig. 2b and Table S13). The consistent top rankings of CB_1_, CB_2_, and GPR55 across multiple centrality metrics reflect their central positions in the curated ECS framework rather than pre-assigned biological weighting. Collectively, these findings illustrate that ECS organization arises from a combination of highly connected receptors, metabolic enzymes, and ligands that integrate receptor-mediated, metabolic, and signaling pathways. By highlighting both canonical and non-canonical nodes, these centrality-based insights provide a framework for prioritizing experimental studies to understand network-level relationships, structural dependencies, and potential targets for pharmacological exploration.

Clustering analysis revealed a modular yet interconnected architecture of the ECS network (Fig. 2c, Figures S3–S4, Tables S6–S11). The network’s connectivity alone was sufficient to uncover biologically coherent functional groupings. Rather than being randomly organized, the network is divided into communities that correspond to key areas such as receptor signaling, endocannabinoid synthesis and degradation, transcriptional regulation, non-canonical signaling mediated by TRP channels, and inflammatory pathways derived from arachidonic acid. This organization highlights that the ECS is structured around functional modules, which remain interconnected through shared ligands, enzymes, and signaling intermediates (Lu and Mackie 2021; Sikyta et al. 2024; Barrie and Manolios 2019; Campolongo and Trezza 2012; Dörnyei et al. 2023; Bernabò et al. 2013; Pistis and Melis 2010; Rezende et al. 2023; Șerban et al. 2025). By delineating distinct yet interacting communities, this framework offers a structured basis for hypothesis-driven investigations and can serve as a guide for future studies focused on specific clusters and their constituent nodes when examining receptor regulation, metabolic flux, transcriptional control, or inflammatory crosstalk. Integrating contextual layers into future network models, such as tissue-specific expression patterns or condition-dependent interactions (Zheng et al. 2019; Berenstein et al. 2016; Zhang et al. 2016; Nasser et al. 2011; Rush and Repsilber 2018; Chen et al. 2021; Li et al. 2024b), could further refine these modules and clarify how ECS connectivity may vary across physiological and pathological states.

Our perturbation analysis indicates that removing highly connected nodes alters the topological structure of the ECS interaction network. Consistent with previous research describing the ECS as a scale-free network (Bernabò et al. 2013), a small subset of highly connected nodes exerts a disproportionate influence on overall connectivity. The targeted removal of these nodes redistributes centrality across the remaining network, increasing the relative importance of some components while decreasing that of others. For instance, after the removal of CB_1_, receptors such as CB_2_ and GPR55 showed increased centrality, whereas certain enzymes like DAGLβ and endocannabinoid-like lipids like LEA showed reduced positional influence. These changes reflect a reconfiguration of the shortest paths and connectivity patterns within the static interaction map, rather than a functional compensation.

Importantly, such perturbation-driven changes identify nodes whose structural roles are contingent upon the presence of dominant hubs and highlight components that assume greater topological prominence under altered configurations. While these results do not imply biological resilience or adaptive signaling responses, they provide a systematic framework for prioritizing hypothesis-driven investigations. Nodes that increase in centrality following hub removal, such as CB_2_ and GPR55, may represent underexplored components whose influence is revealed under perturbed conditions. Conversely, nodes that exhibit substantial decreases in centrality, like DAGLβ and LEA, may reflect interaction patterns highly dependent on specific connectors. Together, these observations offer a network-based strategy for selecting candidate proteins or ligands for experimental validation, mechanistic study, or therapeutic exploration within the broader context of ECS-related signaling and cross-talk (Zheng et al. 2019; Nada et al. 2024; Carels et al. 2023; Ruffner et al. 2007; Tiwari and Dwivedi 2019).

GPR55 consistently ranked among the top nodes across multiple centrality measures, indicating strong structural prominence within the ECS interaction network. In the combined network comprising 80 nodes, GPR55 ranked among the top five nodes for betweenness (5th), closeness (1st), degree (5th), and eigenvector centrality (5th), exceeding the rankings of other non-canonical GPCRs such as GPR18 and GPR119, which were placed substantially lower across these measures (Table S14). These patterns were even more pronounced in the protein–protein network (40 nodes), where GPR55 ranked first in both closeness and eigenvector centrality and third in degree centrality (Table S14). Its normalized eigenvector centrality score approached 1.0, reflecting strong connectivity to other highly ranked nodes within the defined ECS network. In this context, high eigenvector centrality indicates that GPR55 is embedded within densely connected regions of the network, emphasizing its topological prominence.

Consistent with these structural observations, perturbation analysis showed that removal of CB_1_ increased GPR55’s relative centrality ranking, indicating that its positional prominence becomes more pronounced under altered network configurations. These shifts reflect the redistribution of centrality within the static interaction map rather than evidence of biological compensation. Together, these findings identify GPR55 as a structurally central component within the curated ECS framework. Independent experimental studies have implicated GPR55 in cannabinoid-responsive signaling and diverse physiological processes such as synaptic signaling and inflammatory responses (Ryberg et al. 2007; Lauckner et al. 2008; Kargl et al. 2012). While the present analysis does not assign functional significance, the consistent topological prominence of GPR55 across network configurations supports its prioritization for further hypothesis-driven experimental investigation within ECS-related signaling contexts.

Although we constructed a systems-level network of the ECS by integrating experimentally validated interactions between proteins and chemical compounds, this network has inherent limitations that restrict functional interpretation. The network captures structural connectivity and organizational patterns, illustrating how key nodes and interactions shape network topology (Fig. 2a and b), how molecular components cluster into coherent communities (Fig. 2c), and how perturbation of specific nodes redistributes centrality and connectivity (Table 1, Figs. 3, 4, 5, 6). However, it does not incorporate interaction directionality, signaling dynamics, or context-dependent regulation, limiting insights to structural topology rather than functional activity.

Looking forward, this framework lays the groundwork for several avenues of future investigation and addresses key gaps in ECS research. Incorporating interaction directionality, experimentally measured interaction strengths, signaling dynamics, and tissue- or context-specific interactions (Zheng et al. 2019; Berenstein et al. 2016; Zhang et al. 2016; Jiang et al. 2022; Cheng et al. 2012; Wuchty 2006; Nasser et al. 2011; Rush and Repsilber 2018) would enable predictive modeling of ECS responses to endogenous ligands, phytocannabinoids, and synthetic compounds, capabilities that remain largely unexplored in current studies (Bernabò et al. 2013; Bernabò et al. 2014; Liu et al. 2024). Complementary network approaches, such as network proximity analysis, could quantify distances between ECS nodes and disease-associated or drug-target modules, revealing potential cross-talk with other signaling pathways and informing therapeutic strategies (Wu et al. 2023b; Han et al. 2024; Brohée et al. 2008; Cheng et al. 2018). Moreover, perturbation- and centrality-driven analyses can prioritize understudied or structurally sensitive nodes, providing a systematic framework for hypothesis-driven experiments to test their roles in ECS connectivity, signaling, and pharmacological modulation. By combining these approaches, future studies could substantially expand both the conceptual and practical impact of ECS network mapping, uncovering non-obvious regulatory components, identifying candidate targets for experimental validation, and guiding the design of interventions that leverage network topology for translational or therapeutic benefit.

## Conclusion

In this study, we characterized the endocannabinoid system (ECS) as an integrated molecular interaction network using computational, topology-driven analyses. Centrality measures revealed structurally prominent canonical and non-canonical receptors, while community detection demonstrated that the ECS is organized into modular yet interconnected clusters corresponding to receptor-mediated signaling and endocannabinoid metabolic processes. Targeted perturbation of highly connected nodes further clarified how topological influence is redistributed within the network, enabling identification of configuration-dependent components that may warrant deeper experimental investigation. Overall, this work establishes a structural foundation for understanding ECS organization and provides a scalable framework for future studies incorporating additional molecular entities, interaction directionality, signaling dynamics, and tissue-specific context to advance mechanistic and translational research.

## Supplementary Information

Supplementary Material 1.

## Data Availability

All data and code used in this study are available in a GitHub repository at https://github.com/aanyashridhar/ECS-Network.

## Abbreviations

ECS: Endocannabinoid system
CNR1: Cannabinoid receptor 1
CNR2: Cannabinoid receptor 2
CB_1_: Cannabinoid receptor type 1
CB_2_: Cannabinoid receptor type 2
TRPV1: Transient receptor potential vanilloid 1
GPR55: G-protein coupled receptor 55
GPR18: G-protein coupled receptor 18
GPR119: G-protein coupled receptor 119
GPCR: G-protein coupled receptor
AEA: Anandamide
2-AG: 2-Arachidonoylglycerol
Δ^9^-THC: Delta-9-tetrahydrocannabinol
THC: Tetrahydrocannabinol
CBD: Cannabidiol
CBN: Cannabinol
CBC: Cannabichromene
CBG: Cannabigerol
THCV: Tetrahydrocannabivarin
CBDV: Cannabidivarin
OEA: Oleoylethanolamide
LEA: Linoleoyl ethanolamide
PGD2: Prostaglandin D2
NADA: N-arachidonoyl dopamine
NAGly: N-arachidonoyl glycine
PEA: Palmitoylethanolamide
PGE2: Prostaglandin 2
PGA2: Prostaglandin A2
LTB4: Leukotriene B4
HETE: Hydroxyeicosatetraenoic acid
ALOX: Arachidonic acid lipoxygenase
NAAA: N-acylethanolamine-hydrolyzing acid amidase
FAAH: Fatty acid amide hydrolase
MAGL: Monoacylglycerol lipase
NAPE-PLD: N-acyl-phosphatidylethanolamine phospholipase D
DAGLα: Diacylglycerol lipase alpha
DAGLβ: Diacylglycerol lipase beta
GRK2: G protein-coupled receptor kinase 2
ABHD6: α/β-Hydrolase domain-containing protein 6
ABHD12: α/β-Hydrolase domain-containing protein 12
FABP: Fatty acid-binding protein
PPARα: Peroxisome proliferator-activated receptor alpha
PTGS2: Prostaglandin-endoperoxide synthase 2
COX-2: Cyclooxygenase-2
PLCε1: Phospholipase C epsilon 1
PLCβ1: Phospholipase C beta 1
Gαi: Inhibitory Guanine nucleotide-binding protein G alpha subunit
Gαi1: Inhibitory Guanine nucleotide-binding protein G alpha subunit 1
Gαi2: Inhibitory Guanine nucleotide-binding protein G alpha subunit 2
Gαi3: Inhibitory Guanine nucleotide-binding protein G alpha subunit 3
PPI: Protein–protein interaction
PCI: Protein–chemical interaction
SMILES: Simplified Molecular-Input Line-Entry System
IUPAC: International Union of Pure and Applied Chemistry
