# Supplementary material for: Integrative network analysis reveals organizational principles of the endocannabinoid system
Source: J Cannabis Res. 2026 May 13;8:83. doi: 10.1186/s42238-026-00447-3 (PMC13344038; doi:10.1186/s42238-026-00447-3)
Supplement: Supplementary file 1 — Supplementary Material 1. [file 42238_2026_447_MOESM1_ESM.pdf]

Integrative Network Analysis Reveals Organizational Principles of the Endocannabinoid System

Aanya Shridhar[1]*, Sugyan Mani Dixit[2]*, Anthony Torres[2], and Reggie Gaudino[2]**

1. South Brunswick High School, Monmouth Junction, NJ, USA
2. Cannabis Research Institute, Discovery Partners Institute, University of Illinois System, Chicago, IL, USA

*Equal contribution

**To whom correspondence should be addressed:

Reggie Gaudino
Cannabis Research Institute
2201 W Campbell Park Drive
Room 115
Chicago, IL 60612
rgaudino@uillinois.edu

Supporting Information

Table S1: Chemical compounds included in the ECS network. List of chemical nodes (40) with compound names and PubChem CIDs used for integration into the protein–chemical and combined interaction network. Full metadata, including IUPAC names, SMILES strings, and RDKit-calculated descriptors, are provided separately at https://github.com/aanyashridhar/ECS-Network.

Chemical	PubChem CID
AEA	5281969
2-AG	5282280
Noladin	5311103
Virodhamine	6443013
NADA	5311436
AA	5283678
OEA	5286420
PEA	10467
LEA	5283387
NAGly	23667530
Δ9-THC	16078
Δ8-THC	644019
THCA	644017
THCV	644011
CBD	6440190
CBDA	644001
CBDV	92012705
CBG	5281963
CBGA	5281962
CBC	5281965
CBN	65156
CBL	5281447
WIN55,212-2	5311105
WIN55,212-3	5311106
CP55,940	443912
HU-210	5311352
HU-211	146660

JWH-018	588025
JWH-073	9850028
JWH-133	86287562
AM251	6918495
SR141716A	3003824
SR144528	5311135
PGE2	5280360
PGD2	5280896
PGA2	5280895
12-HETE	444899
15-HETE	5280734
5-HPETE	5282820
LTB4	5280934

Table S2: Proteins included in the ECS network. List of protein nodes (40) with protein names and UniProt IDs used for integration into the protein–protein and combined interaction network. Full metadata, including full names, protein types, sub-cellular locations, structural annotations, PDB IDs, amino acid sequences, and evidence codes, are provided separately at https://github.com/aanyashridhar/ECS-Network.

Protein	UniProt ID
CNR1	P21554
CNR2	P34972
GPR55	Q9Y2T6
GPR18	Q14330
GPR119	Q8TDV5
TRPV1	Q8NER1
TRPV2	Q9Y5S1
TRPV3	Q8NET8
TRPV4	Q9HBA0
PPARA	Q07869
PPARD	Q03181
PPARG	P37231
NR1I2	O75469
NAPEPLD	Q6IQ20
PLCB1	Q9NQ66
PLCE1	Q9P212
DAGLA	Q9Y4D2
DAGLB	Q8NCG7
FAAH	O00519
NAAA	Q02083
MGLL	Q99685
ABHD6	Q9BV23
ABHD12	Q8N2K0
PTGS2	P35354
ALOX5	P09917
ALOX12	P18054
ALOX15	P16050
FABP1	P07148

FABP3	P05413
FABP5	Q01469
FABP7	O15540
HSPA1A	P0DMV8
SLCO2B1	O94956
SLC22A5	O76082
GNAI1	P63096
GNAI2	P04899
GNAI3	P08754
ARRB1	P49407
GRK2	P25098
AKT1	P31749

Table S3: Protein–protein interactions included in the ECS network. List of protein–protein interactions (92) with interacting protein pairs and confidence scores (thresholded at 0.7) used for integration into the protein–protein and combined interaction network.

Interacting Protein A	Interacting Protein B	Confidence
GRK2	ARRB1	0.998
CNR1	GNAI1	0.995
GNAI2	CNR1	0.993
GNAI2	GNAI3	0.992
ARRB1	AKT1	0.99
FABP5	PPARD	0.988
GNAI3	GNAI1	0.984
CNR1	GPR55	0.983
CNR1	CNR2	0.981
GNAI2	GNAI1	0.975
FAAH	MGLL	0.974
PTGS2	ALOX5	0.973
FAAH	CNR1	0.972
FABP1	PPARA	0.964
ALOX15	PTGS2	0.963
ABHD6	ABHD12	0.949
ALOX12	PTGS2	0.947
MGLL	CNR1	0.941
CNR1	GNAI3	0.933
FAAH	NAPEPLD	0.931
PLCB1	GNAI3	0.929
FABP1	FABP3	0.929
PLCB1	GNAI1	0.928
GNAI2	PLCB1	0.926
GPR119	GPR55	0.922
TRPV1	GPR55	0.922
MGLL	NAPEPLD	0.92
DAGLA	DAGLB	0.915
ALOX15	ALOX5	0.914
ALOX12	ALOX5	0.914

CNR2	GNAI1	0.909
ALOX12	ALOX15	0.906
MGLL	ABHD6	0.904
FAAH	DAGLA	0.898
DAGLA	NAPEPLD	0.894
FAAH	FABP5	0.891
DAGLB	NAPEPLD	0.89
DAGLA	MGLL	0.887
PPARD	PPARA	0.887
FAAH	CNR2	0.886
FAAH	ABHD6	0.881
GPR119	GPR18	0.868
FAAH	TRPV1	0.855
FAAH	NAAA	0.85
FAAH	GPR55	0.848
FAAH	DAGLB	0.848
MGLL	ABHD12	0.839
FAAH	FABP7	0.839
FAAH	ABHD12	0.831
DAGLA	ABHD6	0.831
MGLL	DAGLB	0.829
PPARG	FABP5	0.828
FAAH	FABP3	0.826
GPR18	CNR2	0.823
ABHD6	NAPEPLD	0.821
DAGLA	CNR1	0.817
GPR18	CNR1	0.816
NAAA	NAPEPLD	0.816
ABHD6	DAGLB	0.812
ABHD12	NAPEPLD	0.803
NAPEPLD	GPR55	0.801
CNR1	NAPEPLD	0.8
GPR18	TRPV1	0.796
CNR2	GPR55	0.793
DAGLA	ABHD12	0.791

PPARG	AKT1	0.79
CNR1	TRPV1	0.781
MGLL	NAAA	0.78
GRK2	AKT1	0.778
MGLL	GPR55	0.776
PTGS2	AKT1	0.775
TRPV4	TRPV1	0.773
NAPEPLD	TRPV1	0.771
GPR18	GPR55	0.767
CNR1	ARRB1	0.757
DAGLB	ABHD12	0.746
PPARA	GPR55	0.746
ARRB1	GPR55	0.744
FAAH	GPR18	0.736
PPARG	PTGS2	0.735
PTGS2	PPARA	0.729
MGLL	TRPV1	0.728
PPARA	TRPV1	0.718
ABHD6	GPR55	0.717
PPARG	PPARD	0.715
FABP5	PPARA	0.708
GPR119	PPARA	0.706
DAGLB	CNR1	0.705
PLCB1	PLCE1	0.705
NAAA	PPARA	0.702
PPARA	AKT1	0.7
DAGLA	GPR55	0.7

Table S4: Protein–chemical interactions included in the ECS network. List of protein–chemical interactions (126) with interacting chemicals and interacting proteins used for integration into the protein–chemical and combined interaction network.

Chemical	Protein
AEA	CNR1
AEA	CNR2
AEA	FAAH
AEA	MGLL
AEA	TRPV1
2-AG	MGLL
2-AG	CNR2
2-AG	CNR1
2-AG	DAGLB
2-AG	DAGLA
2-AG	GPR119
2-AG	FAAH
NADA	TRPV1
NADA	FAAH
AA	PTGS2
AA	ALOX5
OEA	GPR119
OEA	NAAA
OEA	CNR2
OEA	PPARA
OEA	TRPV1
OEA	PPARD
OEA	CNR1
PEA	NAAA
LEA	TRPV1
LEA	CNR1
NAGly	TRPV1
NAGly	FAAH
Δ9-THC	GPR18
Δ9-THC	PPARD

Δ8-THC	GPR18
Δ8-THC	PPARD
THCA	ALOX15
THCV	CNR2
THCV	TRPV1
THCV	TRPV2
THCV	TRPV4
THCV	CNR1
THCV	NAAA
THCV	GPR55
THCV	TRPV3
THCV	DAGLA
CBD	PPARG
CBD	NR1I2
CBD	CNR1
CBD	TRPV1
CBD	PTGS2
CBD	CNR2
CBD	DAGLA
CBD	NAAA
CBD	GPR55
CBDA	ALOX15
CBDV	GPR55
CBDV	CNR2
CBDV	TRPV2
CBDV	DAGLA
CBDV	TRPV1
CBDV	TRPV4
CBDV	NAAA
CBDV	TRPV3
CBDV	CNR1
CBG	PPARG
CBG	TRPV2
CBG	TRPV1
CBG	CNR2

CBG	CNR1
CBG	DAGLA
CBG	TRPV3
CBG	NAAA
CBG	TRPV4
CBGA	DAGLA
CBGA	NAAA
CBGA	CNR1
CBGA	ALOX15
CBC	TRPV3
CBC	TRPV4
CBC	CNR2
CBC	DAGLA
CBC	NAAA
CBC	TRPV2
CBC	PPARG
CBC	TRPV1
CBC	CNR1
CBN	CNR2
CBN	CNR1
CBN	TRPV1
CBN	TRPV2
CBN	NAAA
CBN	TRPV4
CBN	DAGLA
CBN	PPARG
CBN	TRPV3
WIN55,212-2	CNR1
WIN55,212-2	CNR2
WIN55,212-3	CNR1
WIN55,212-3	CNR2
CP55,940	CNR1
CP55,940	CNR2
HU-210	CNR2
HU-210	CNR1

HU-211	CNR1
HU-211	CNR2
JWH-018	CNR2
JWH-018	CNR1
JWH-073	CNR2
JWH-133	CNR2
JWH-133	CNR1
JWH-133	TRPV1
AM251	CNR1
AM251	TRPV1
AM251	GPR55
AM251	CNR2
SR141716A	CNR1
SR144528	CNR2
SR144528	CNR1
PGE2	PTGS2
PGD2	PPARG
PGD2	PPARA
PGA2	PTGS2
12-HETE	ALOX5
12-HETE	ALOX15
12-HETE	ALOX12
12-HETE	PTGS2
12-HETE	PPARA
15-HETE	ALOX5
LTB4	ALOX5

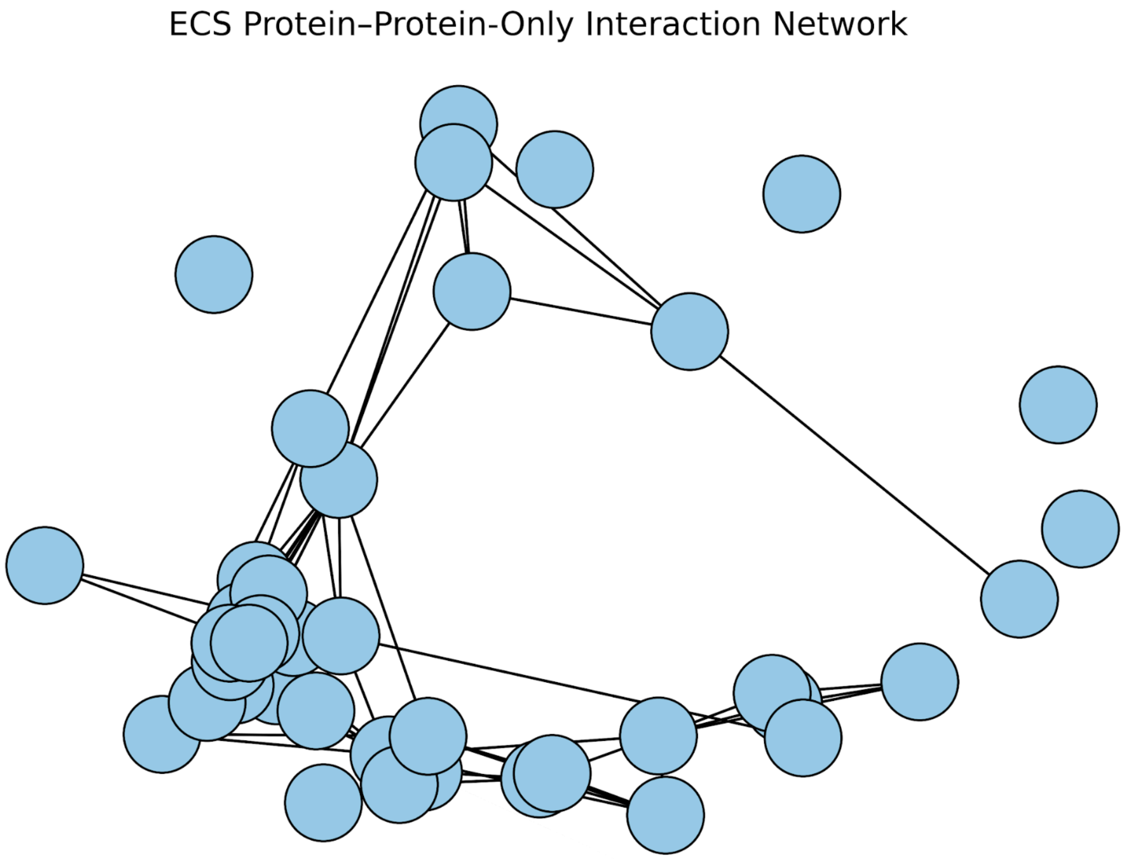

Figure S1: Protein–protein interaction network. Visualization of the protein–protein only network showing proteins connected through documented interactions. The network has 40 nodes and 92 edges.

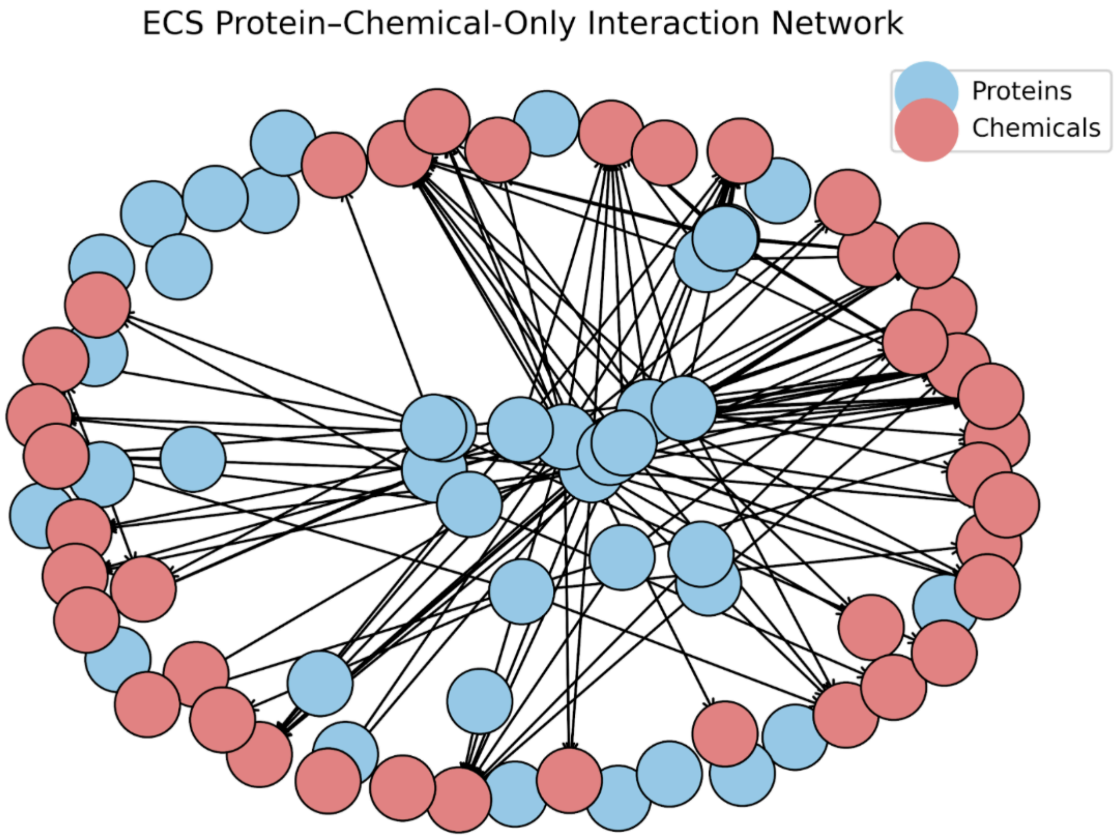

Figure S2: Protein–chemical interaction network. Visualization of the protein–chemical only network (with protein–protein interactions filtered out) showing proteins (blue) and chemicals (red) connected through documented interactions. The network has 40 nodes and 126 edges.

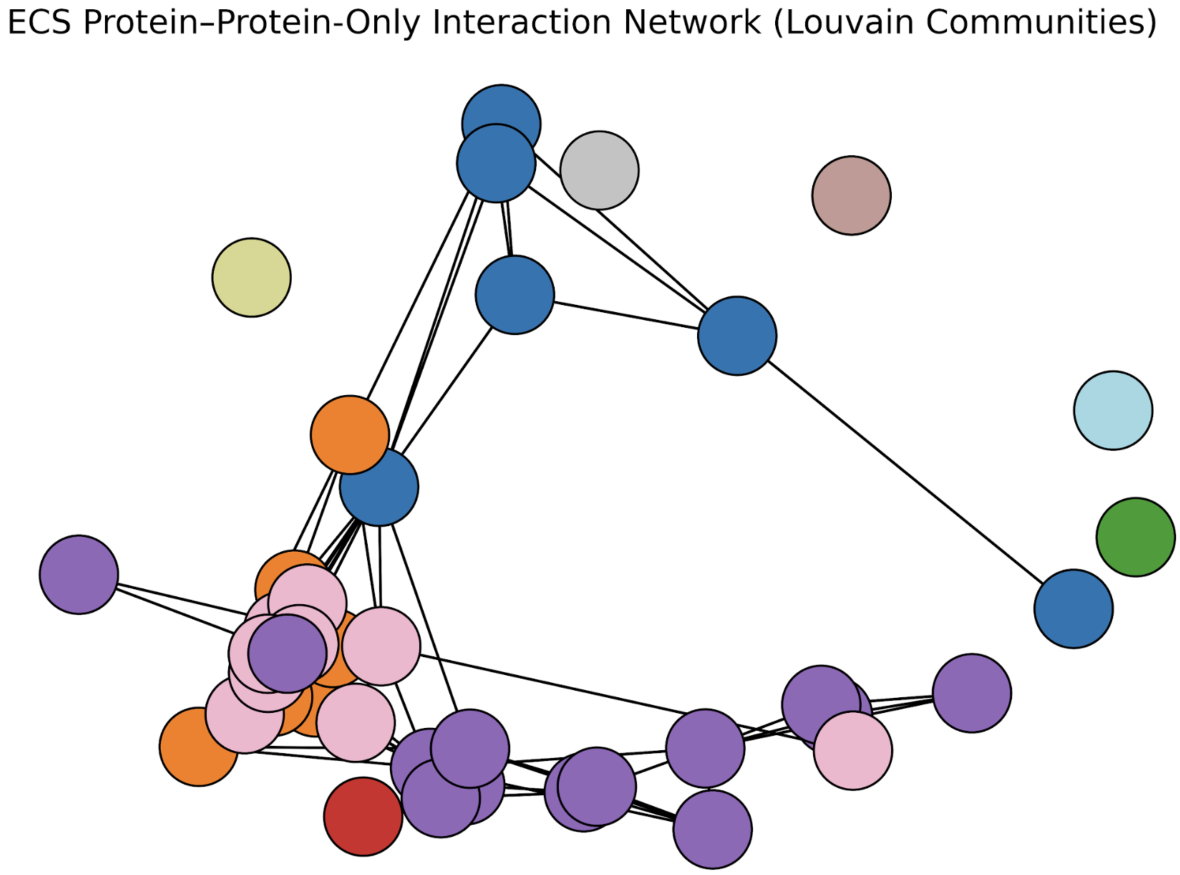

Figure S3: Protein–protein interaction network Louvain communities from clustering analysis. Louvain community detection applied to the protein–protein only network, partitioning the nodes into modular clusters (denoted by distinct colors) based on interaction density, which highlights potential functional or mechanistic groupings within ECS proteins.

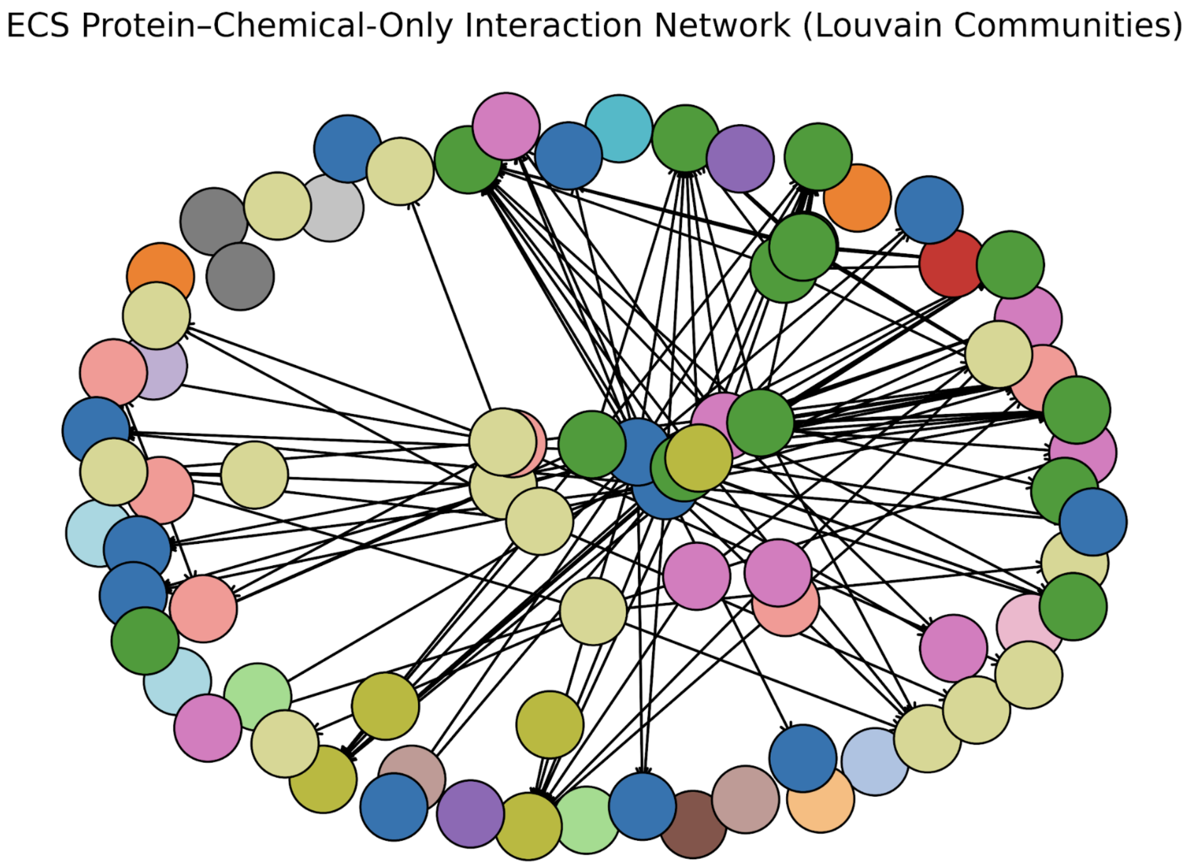

Figure S4: Protein–chemical interaction network Louvain communities from clustering analysis. Louvain community detection applied to the protein–chemical only network (with protein–protein interactions filtered out), partitioning the nodes into modular clusters (denoted by distinct colors) based on interaction density, which highlights potential functional or mechanistic groupings within ECS proteins and chemicals.

Table S5: Louvain community assignments for the combined interaction network. List of proteins and chemicals and their assigned Louvain communities.

Node	Louvain Community
CNR1	0
CNR2	0
PLCE1	0
PLCB1	0
AEA	0
GNAI3	0
GNAI1	0
GNAI2	0
WIN55,212-3	0
WIN55,212-2	0
JWH-133	0
JWH-073	0
JWH-018	0
HU-211	0
HU-210	0
CP55,940	0
SR141716A	0
AM251	0
SR144528	0
SLCO2B1	1
FAAH	2
NAPEPLD	2
DAGLB	2
ABHD12	2
2-AG	2
FABP7	2
ABHD6	2
MGLL	2
GPR55	2
DAGLA	2
SLC22A5	3

THCV	4
CBC	4
CBN	4
CBDV	4
PEA	4
CBG	4
TRPV2	4
TRPV3	4
NADA	4
TRPV4	4
NAAA	4
LEA	4
NAGly	4
TRPV1	4
GRK2	5
Δ8-THC	5
ARRB1	5
AKT1	5
OEA	5
GPR119	5
GPR18	5
PPARA	5
FABP3	5
PGD2	5
Δ9-THC	5
PPARG	5
PPARD	5
FABP1	5
FABP5	5
Noladin	6
CBD	7
NR1I2	7
PTGS2	8
ALOX15	8
ALOX12	8

PGE2	8
THCA	8
AA	8
CBDA	8
ALOX5	8
CBGA	8
12-HETE	8
15-HETE	8
LTB4	8
PGA2	8
Virodhamine	9
CBL	10
5-HPETE	11
HSPA1A	12

Table S6: Louvain community summary metrics for the combined interaction network. Report of overall network modularity, the total number of detected communities, and community size statistics.

Metric	Value
Overall Modularity	0.4277
Number of Communities	13
Largest Community Size	19
Smallest Community Size	1

Table S7: Louvain community assignments for the protein–protein interaction network. List of proteins and their assigned Louvain communities.

Protein	Louvain Community
CNR1	0
PLCB1	0
PLCE1	0
GNAI2	0
GNAI1	0
GNAI3	0
GPR119	1
CNR2	1
TRPV4	1
TRPV1	1
GPR55	1
GPR18	1
TRPV2	2
TRPV3	3
PPARG	4
PPARA	4
FABP1	4
ALOX5	4
FABP5	4
FABP3	4
ALOX12	4
PPARD	4
ALOX15	4
PTGS2	4
ARRB1	4
GRK2	4
AKT1	4
NR1I2	5
ABHD12	6
ABHD6	6
NAPEPLD	6

DAGLA	6
NAAA	6
FABP7	6
FAAH	6
DAGLB	6
MGLL	6
SLC22A5	7
SLCO2B1	8
HSPA1A	9

Table S8: Louvain community summary metrics for the protein–protein interaction network. Report of overall network modularity, the total number of detected communities, and community size statistics.

Metric	Value
Overall Modularity	0.4207
Number of Communities	10
Largest Community Size	13
Smallest Community Size	1

Table S9: Louvain community assignments for the protein–chemical interaction network. List of proteins and chemicals and their assigned Louvain communities.

Node	Louvain Community
CNR1	0
CNR2	0
WIN55,212-3	0
WIN55,212-2	0
JWH-018	0
HU-211	0
HU-210	0
CP55,940	0
SR141716A	0
SR144528	0
JWH-073	0
GNAI1	1
GNAI2	2
GNAI3	3
ARRB1	4
GRK2	5
CBG	6
CBN	6
CBC	6
THCV	6
CBDV	6
TRPV4	6
PPARG	6
PGD2	6
AM251	6
PEA	6
GPR55	6
TRPV3	6
TRPV2	6
NAAA	6
AKT1	7

Noladin	8
Virodhamine	9
PPARD	10
GPR18	10
GPR119	10
Δ8-THC	10
OEA	10
Δ9-THC	10
CBL	11
5-HPETE	12
NAPEPLD	13
PLCB1	14
PLCE1	15
FABP7	16
NADA	17
AEA	17
TRPV1	17
FAAH	17
MGLL	17
NAGly	17
LEA	17
JWH-133	17
HSPA1A	18
SLCO2B1	19
ABHD6	20
ABHD12	21
DAGLB	22
2-AG	22
DAGLA	22
NR1I2	22
CBD	22
ALOX12	23
ALOX15	23
PPARA	23
PTGS2	23

ALOX5	23
PGE2	23
AA	23
CBGA	23
THCA	23
CBDA	23
PGA2	23
12-HETE	23
15-HETE	23
LTB4	23
SLC22A5	24
FABP1	25
FABP3	26
FABP5	27

Table S10: Louvain community summary metrics for the protein–chemical interaction network. Report of overall network modularity, the total number of detected communities, and community size statistics.

Metric	Value
Overall Modularity	0.3969
Number of Communities	28
Largest Community Size	14
Smallest Community Size	1

Table S11: Protein–protein interaction network centrality metrics. Top five nodes ranked by centrality measures in the protein–protein only network. Full rankings and centrality metric calculations can be found at https://github.com/aanyashridhar/ECS-Network.

	Betweenness	Closeness	Degree	Eigenvector
1	CNR1	GPR55	FAAH	GPR55
2	PPARA	TRPV1	CNR1	TRPV1
3	PTGS2	AKT1	GPR55	GNAI1
4	ARRB1	NAPEPLD	NAPEPLD	AKT1
5	GPR18	PPARA	MGLL	NAPEPLD

Table S12: Protein–chemical interaction network centrality metrics. Top five nodes ranked by centrality measures in the protein–chemical only network. Betweenness centrality is excluded in the protein–chemical network as this centrality relies on shortest path calculations between nodes, which are not well-defined in networks where chemicals only connect to proteins. Full rankings and centrality metric calculations can be found at https://github.com/aanyashridhar/ECS-Network.

	Closeness	Degree	Eigenvector
1	CBN	CNR1	CBN
2	CBC	CNR2	CBC
3	CBD	TRPV1	CBD
4	THCV	CBDV	THCV
5	CBG	CBN	CBG

Table S13: Comparative centrality rankings of G-protein coupled proteins, GPR55, GPR18, and GPR119. Rankings of three non-canonical cannabinoid receptors across the combined ECS network and the protein–protein only network showing GPR55 outperforming GPR18 and GPR119 in nearly every metric, with the exception of betweenness centrality in the protein–protein only network. Unranked indicates a centrality value of zero, meaning the node had no measurable contribution for that metric. Full rankings and centrality metric calculations can be found at https://github.com/aanyashridhar/ECS-Network.

Network	Centrality	**GPR55**	**GPR18**	**GPR119**
Combined	Betweenness	5	8	Unranked
Combined	Closeness	1	51	Unranked
Combined	Degree	5	19	29
Combined	Eigenvector	5	56	63
Protein–Protein	Betweenness	Unranked	5	Unranked
Protein–Protein	Closeness	1	18	Unranked
Protein–Protein	Degree	3	11	24
Protein–Protein	Eigenvector	1	20	27
